# An Analysis of Scalp Thickness and Other Novel Risk Factors for Deep Brain Stimulator Infections

**DOI:** 10.7759/cureus.792

**Published:** 2016-09-20

**Authors:** Nicholas Brandmeir, Elena Nehrbass, James McInerney

**Affiliations:** 1 Department of Neurosurgery, Penn State Milton S Hershey Medical Center

**Keywords:** deep brain stimulator infection, scalp thickness, infection, dbs infection, dbs

## Abstract

Introduction: Deep brain stimulator (DBS) infections are a persistent problem for patients undergoing this procedure. They may require further surgery, treatment with antibiotics, or even removal of the device. To date, no consensus exists on the best practices to avoid DBS infections or what factors predispose patients to an eventual infection. The goal of this study was to examine several patient factors for association with DBS infection.

Methods: A single-center, single-surgeon quality improvement database was queried. All patients who experienced an infection were identified. The primary variable analyzed was scalp thickness. Other pre-specified, secondary variables included routine intraoperative cultures, operative time, diagnosis, and age.

Results: None of the independent variables examined were significantly associated with DBS infections. Only two of the 46 infections qualified as surgical site infections as defined by the Centers for Disease Control.

Conclusion: DBS infections are independent of all of the predictor variables analyzed. Surgical site infections, according to traditional definitions, are not the optimal definition for evaluating DBS infections/erosions. New studies must examine new variables that are not routinely gathered in this population. Also, because of the rare event rates and difficulty in randomizing patients to exposures, a large, multicenter registry may be the optimal study design to solve this clinical problem.

## Introduction

Deep brain stimulation (DBS) has become the most widely used treatment modality for otherwise treatment refractory movement disorders [1-2]. Although the risk profile is very low, hardware complications are the most common complaint among patients with DBS [3]. Among hardware complications, infection and skin erosion are a particular problem [3-6]. This is because infections, unlike some other hardware-related complications, often require further surgery to treat, can jeopardize re-implantation should a lead removal be required, and can lead to significant patient discomfort. Also, infections can occur after the patient is receiving the benefit of the DBS therapy and removal of hardware at that stage or a repeat operation is often particularly frustrating to the patient [7]. Finally, while rare, DBS infections can also be life-threatening intracerebral infections [8].

There is also conflicting data about the true rate and definition of infections in DBS [9]. Some large series have reported zero infections while others have reported infection rates approaching 10% [4, 10]. Some authors have considered erosion/wound dehiscence as part of a spectrum of infection while others have considered erosion to be a unique complication separate from infection [2-3, 5, 7, 10-14].

Previous studies have tried to quantify the risk factors associated with DBS infections. Different independent variables considered have included age, diabetes, gender, Unified Parkinsons Disease Rating Scale (UPDRS), and hypertension [3, 15]. None of these proposed predictors demonstrated any association with DBS infections. Another study found that the length of surgery, operating surgeon, scalp erosion, and the number of people present in the operating room were associated with an infection within 30 days of the initial operation [12].

Perhaps unsurprisingly, the treatment and prevention strategies for DBS infection also vary widely across the literature [9]. Some authors recommend only preoperative antibiotics [11], while others recommend that antibiotics be given postoperatively as well [16]. Some authors have recommended implantation of the hardware as deep in the soft tissue as possible, although none of these studies showed a statistically significant benefit to this practice [11, 17-18]. One study demonstrated a small, statistically significant decrease in the risk of infection when leads were implanted in a one-stage operation rather than a two-stage operation (4.2% vs. 15.3%, p < 0.0003); however, it is worth noting that in this series, two-stage procedures were often accompanied by a trial of externalized stimulator leads [13]. Other papers have compared curvilinear incisions to straight incisions as a means of preventing infections, but again, these maneuvers did not provide a statistically significant benefit [13, 19]. 

Many of the strategies of treating and preventing DBS infections focus on preserving skin health and integrity [13, 17, 19-22]. Even though this makes good sense from first principles, no study has yet looked at an objective association between scalp health and later infection. Our hypothesis was that a thinner scalp would predispose patients undergoing DBS to infection.

## Materials and methods

**Figure 1:**
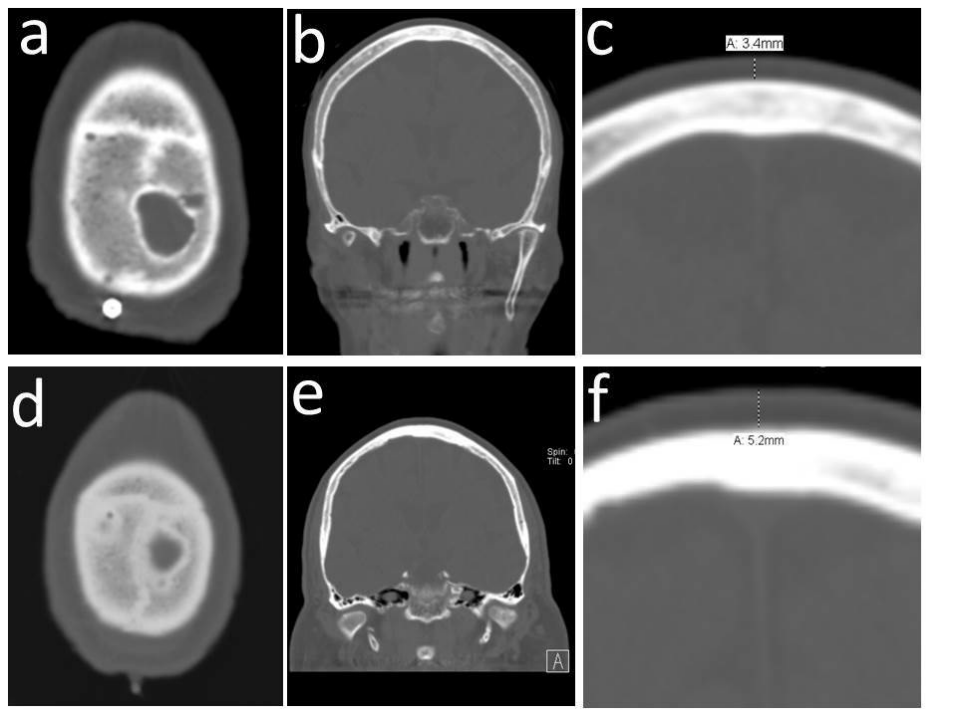
Measurement of Scalp Thickness in Two Patients (a-c and d-f) a,d: Axial CT scans showing typical coronal suture imaging. b, e: Coronal CT reconstructions at the level of the coronal suture for both patients. c, f: Examples of the measurement of scalp thickness at the midline over the coronal suture.

This study was a review of a prospectively gathered database of patients who underwent DBS therapy at a single surgeon practice in an academic center from the years 2008 to 2015. Scalp thickness was measured by measuring the scalp with imaging software (IDX, General Electric, Schenectady, NY, USA) in the midline over the coronal suture (Figure 1) by authors NB and EN. The precision of this measurement was determined by calculating a percent agreement. Measurements were said to agree if they were within 0.5 mm or about 1 pixel on our imaging software.

All surgeries were carried out by the same attending surgeon using a platform stereotactic system (StarFix, FHC, Inc., Bowdoin, ME, USA). All surgeries were done in a two-stage fashion with Stage one consisting of bilateral lead placement and Stage two consisting of placement of a dual channel generator and connection to the leads via lead extensions. All DBS systems placed were Medtronic hardware (Medtronic, Inc., Minneapolis, MN, USA). All patients in this cohort were treated with pre- and postoperative antibiotics according to the same protocol.

Scalp thickness was the primary, pre-specified independent variable of the analysis; however, other important variables in predicting infections were considered. Patient age, as well as the duration of lead placement and generator placement surgeries, were the independent variables considered. It is also our practice to obtain routine cultures of the skull/burr hole just prior to closure of the lead implantation surgery. The positivity of these cultures was also considered as an independent variable in predicting lead infection. We also considered the primary diagnosis as an independent risk factor.

Infection and erosion were considered as one common outcome using the Centers for Disease Control (CDC) definition of surgical site infections (SSI) as the clinical definition of infection [22]. Early infections were those infections occurring within 30 days of the placement of the stimulating electrodes (a traditional SSI as defined by the CDC) and late infections were any that met the definition after this point.

Statistical analyses were carried out using Microsoft Excel (Microsoft, Inc., Redmond, WA, USA) and SAS software (SAS Institute Inc., Cary, NC, USA). Significance was set at an alpha of 0.05 with a Bonferroni correction for multiple comparisons [6]. The final alpha required for significance was 0.0083. Continuous variables were compared using a T-test while categorical variables were compared using a Chi-squared test.

Study data were collected and managed using research electronic data capture (REDCap) tools hosted at the institution. REDCap is a secure, web-based application designed to support data capture for research studies [23].

This study was conducted as a review of an existing quality improvement database and, as such, was exempt from IRB approval. Informed consent was waived because the study used existing data.

## Results

This study included 384 patients. One hundred fifty-three patients were excluded from the analysis because imaging characteristics prevented accurate measurement of their scalp thickness at the coronal suture. Some patients lacked coronal reconstructions in their images while others had significantly ossified sutures that prevented accurate identification for measurement purposes (Figure 1). Of those that had available scalp thickness data, 214 had an operative time recorded for their lead placement surgery and 213 had an operative time recorded for their generator placement/lead connection surgery. Every patient had accurate age data available. Two hundred fifteen patients had routine intraoperative cultures available from their lead placement surgery. Basic demographic statistics of the patient population is summarized in Table 1. The surgical site infection rate by CDC criteria was 0.9% (2/219). The overall rate of infection/erosion in our total series, however, was 21% (46/219). Our protocol for the management of DBS infections is to attempt conservative therapy with skin care and oral antibiotics followed by revision surgery and explant if these maneuvers fail. Table 2 details the success rates of each treatment strategy. Nineteen out of 46 (41%, 8.7% of the total) infected patients were able to have their hardware salvaged with antibiotic treatment alone or with a skin revision surgery and antibiotic therapy; the remainder of the patients (59%, 12% of the total) required explantation of their systems. Percent agreement between the authors for measurement of scalp thickness was 80.5%, which is sufficient to ensure good agreement.

**Table 1:** General Patient Cohort Characteristics Stage 1 refers to lead placement and Stage 2 refers to the placement of the generator and connection via lead extensions. PD = Parkinsons disease; ET = essential tremor; OCD = obsessive compulsive disease; MS = multiple sclerosis; Dys = Dystonia; OR = operating room

Mean Age		66 yrs
% with Diagnosis	PD	62
	ET	32
	Dyst	3
	MS	3
	OCD	<1
Mean OR Time Stage 1		170 min
Mean OR Time Stage 2		54 min
Mean Scalp Thickness		5.03 mm

**Table 2:** Details of Patients Suffering a DBS Infection/Erosion Early surgical site infections (SSI) were infections that met CDC SSI infection criteria within 30 days of stimulating electrode implantation. DBS - deep brain stimulation; OR = operating room

Mean days until infection		165
Median number of OR		1 (0-4)
Total number of infections		46 (100%)
	Early SSI	2 (4%)
Treatments	Antibiotics alone	2(4%)
	Revised and retained	17 (37%)
	Revised and then removed	15 (33%)
	Removed at 1^st^ surgery	12 (26%)

Summary statistics comparing the likelihood of infection based on each of the independent predictors is available in Table 3. In short, none of our pre-specified predictors were associated with infection.

**Table 3:** Effect of Independent Variables on DBS Infection Scalp thickness, operating room (OR) times, and age are compared with T-test and differences are reported with absolute mean differences and p-values. Diagnosis and culture positivity are compared by means of a Chi-squared test. Results are reported as a relative risk with 95% confidence interval and p-value. Stage 1 refers to the placement of bilateral leads. Stage 2 refers placement of the generator and connecting the leads with lead extensions. Relative risk (RR) values are not available for diagnosis because it was not quantifiable in a 2 x 2 table.

	RR/mean difference	p-value
Scalp Thickness	0.1	0.607
Stage 1 OR Time	6.6	0.263
Stage 2 OR Time	5.5	0.018
Age	1.1	0.549
Diagnosis		0.657
Culture +	1.410 (0.828 - 2.402)	0.206

## Discussion

This report is a large single-surgeon series of all patients presenting at an academic center undergoing DBS surgery. It is the first to examine the relation of several important factors to DBS infection. Skin health (as measured by scalp thickness over the coronal suture), routine intraoperative culture to evaluate contamination and flora, the role of primary diagnosis as a risk for infection, and the length of the entire operation, both Stage 1 and Stage 2. We also evaluated age as an independent variable for infection. Another study has evaluated age as an independent variable [15], but our study treated age as a continuous rather than a dichotomous variable, which is more likely to show the true effect of age on the pathology. 

Also, because the patients in this series all had a minimum of six months of follow-up and many with years of follow up, the problem of delayed infections was accurately captured and evaluated. This is more clinically relevant information because patients contemplating DBS surgery want to know about the life of the device and how likely they are to need more surgery rather than how likely they are to have an infection within 30 days. Interestingly, although we identified 46 patients who had a device-related infection/erosion, only two of these were within 30 days. This is important because CDC SSI criteria almost certainly miss the vast majority of device-related infections in DBS and certainly did in our patient cohort. 

Other studies have evaluated the risk of DBS infections in the past but few have done so with more than a simple case series [3, 7, 10-11, 16, 24-26]. In several of those studies that sought a correlation of some risk factor with eventual infection, no risk factors have been identified that are associated with infection [13, 15, 19]. Tolleson, et al. identified that the operating surgeon, the number of people present in the operating room, skin erosion, and ‘surgical incision opening time’ were independently associated with infection [12]. That study focused on 30-day infection rates rather than overall infection rates and considered skin erosion and infection as different outcomes while, in our report, we considered them part of the same clinical spectrum. Another study compared one-stage procedures to two-stage procedures for DBS placement and one-stage procedures were associated with a lower rate of infection [13]. Our study was based on a quality improvement database and our practice is to do staged procedures, so this comparison was unavailable to us in this data set. 

For some of the variables explored, the difference in the means did not meet with any biological plausibility (for instance, a shorter Stage 2 operating time was associated with a higher risk of infection). For this reason, these differences are almost certain to be statistical anomalies and this is in agreement with the presented p-value. This was the case for age, scalp thickness, and Stage 2 operating time. For Stage 1 operating time and intraoperative routine cultures, the trend of the difference did seem plausible. We performed a power calculation based on these differences and the assumption that they may represent a real difference between the groups to determine the size of the prospective trial necessary to detect the effect. A trial necessary to detect the effect of operative time in Stage 1 would require 420 patients in each arm and 510 would be required to determine the true impact of intraoperative cultures. The extreme difficulty in designing a trial to evaluate these factors and the amount of time required to capture data on this number of patients may very well render a single center study an impossibility. For these reasons, the most efficient way to answer this question is through the use of large, multicenter, prospectively designed registries rather than continued single-center retrospective analyses or randomized controlled trials. 

This study has several limitations. Chiefly, because it is a single institution, single surgeon series, and important variables like operative technique, institutional decontamination/sterility procedures, patient referral patterns, and demographics cannot be evaluated. Also, because this is a retrospective study, data that wasn’t gathered is not available for analysis. This could include potentially important predictors of infection like MRSA status, tobacco use, diabetes, nutritional status, and surgical site infection just to name a few. These factors may prove to be important but were beyond the scope of our study. Another limitation is the significant number of patients that were screened in the database but excluded from analysis. These patients tended to be from earlier surgeries and the lack of available information is secondary to the lack of technological advances available during the time of the surgery (lack of electronic medical records, poor CT scan quality, lack of imaging archival, etc.). Thus, this exclusion is almost certainly non-random, but it is unclear to what extent, if any, this patient loss affects the results. Unfortunately, this remains an insurmountable problem of retrospective studies and must be accounted for when generalizing this data. 

This is an analysis of a large, prospectively gathered, single-surgeon, quality improvement database of all patients undergoing DBS surgery at an academic center to evaluate risk factors for infection. None of the independent variables analyzed were found to have a statistically significant effect. Future studies addressing the risk of infections in DBS surgery should examine larger, prospectively designed registries. Also, new variables should be investigated, especially those that are not routinely gathered in this patient population. Some predictors that may be related are nutritional status, tobacco use, and diabetes. It is also possible that DBS infections experienced in patients with modern surgical techniques are largely stochastic and that no accurate predictors exist for infection risk.

## Conclusions

Age, the length of operation, primary diagnosis, routine intraoperative cultures, and scalp thickness are not predictive of eventual DBS infection/skin erosion. The CDC 30-day definition of SSI likely leads to underreporting of infection in DBS patients.
